# Assessment of taxonomic and genetic diversity of wood mice of the genus Apodemus in the steppe zone of the Central Ciscaucasia

**DOI:** 10.18699/vjgb-26-93

**Published:** 2026-10

**Authors:** F.A. Tembotova, A.Kh. Amshokova, M.S. Gudova, E.P. Kononenko

**Affiliations:** Tembotov Institute of Ecology of Mountain Territories of the Russian Academy of Sciences, Nalchik, Russia; Tembotov Institute of Ecology of Mountain Territories of the Russian Academy of Sciences, Nalchik, Russia; Tembotov Institute of Ecology of Mountain Territories of the Russian Academy of Sciences, Nalchik, Russia; Tembotov Institute of Ecology of Mountain Territories of the Russian Academy of Sciences, Nalchik, Russia

**Keywords:** Central Ciscaucasia, steppe zone, cytochrome b, genus Apodemus, genetic diversity, Центральное Предкавказье, степная зона, цитохром b, род Apodemus, генетическое разнообразие

## Abstract

Steppe ecosystems are among the most heavily modified habitats due to anthropogenic pressure and rapid land-use changes. Over the course of several decades of the 20th century, the biogeocenoses of the steppe plains and a significant portion of the forest-steppe of the Central Ciscaucasia have almost completely lost their original appearance. Anthropogenic impact resulted in the reduction of suitable habitats, their fragmentation, and so on. It is known that prolonged isolation of a population, caused by natural or anthropogenic habitat fragmentation, can lead to changes in the genetic structure of the population, specifically to a decrease in genetic diversity and a reduction in the total effective population size, which is particularly detrimental to small populations. The relevance of this study is due to the lack of current data on the species composition, genetic structure, genetic diversity, habitat distribution, and abundance of Apodemus wood mice in the steppe zone of the Central Ciscaucasia. Based on an analysis of the mitochondrial DNA cytochrome b (cyt b) gene fragment variability, the species composition and genetic diversity of sibling species of wood mice of the genus Apodemus have been studied for the first time in the steppe zone of the Central Ciscaucasia. Molecular genetic analysis revealed that the genus Apodemus is represented by three species: A. uralensis, A. witherbyi, and A. flavicollis. A. flavicollis and A. witherbyi are described for the first time in this area. Of the three taxa, A. uralensis exhibits the highest level of genetic diversity. Molecular dating results suggest that of the three species, A. uralensis was the first to colonize the Central Ciscaucasia in two stages: approximately 370–350 thousand years ago and approximately 256 thousand years ago. Subsequently, A. witherbyi entered the area in two stages: approximately 166–154 thousand years ago and 125–115 thousand years ago. A. flavicollis is the youngest inhabitant of the Central Ciscaucasia, with an evolutionary age of approximately 100 thousand years. This study identifies the distribution patterns and landscape-biotopic distribution of representatives of the genus Apodemus in the study areas of the Central Ciscaucasia.

## Abstract (ru)

Степные экосистемы – одни из наиболее измененных местообитаний из-за антропогенного давления и быстрых изменений в землепользовании. За несколько десятилетий XX в. биогеоценозы степных равнин и значительной части лесостепи Центрального Предкавказья почти полностью утратили свой первоначальный облик. Результатом антропогенного воздействия являются сокращение пригодных местообитаний, их фрагментация и т. д. При этом известно, что длительная изоляция популяции, вызванная естественной или антропогенной фрагментацией среды обитания, может привести к изменению генетической структуры популяции, в частности к уменьшению генетического разнообразия и сокращению общей эффективной численности популяции, что особенно губительно для популяции небольшого размера. Актуальность проведения настоящего исследования связана с отсутствием современных данных, касающихся видового состава, генетической структуры, генетического разнообразия, биотопического распространения и численности представителей лесных мышей рода Apodemus в условиях степной зоны Центрального Предкавказья. На основании анализа изменчивости фрагмента гена цитохрома b (сyt b) митохондриальной ДНК впервые изучены видовой состав и генетическое разнообразие видов-двойников лесных мышей рода Apodemus в условиях степной зоны Центрального Предкавказья. По результатам молекулярно-генетического анализа род Apodemus представлен тремя видами: A. uralensis, A. witherbyi и A. flavicollis. На рассматриваемой территории A. flavicollis и A. witherbyi описаны впервые. Из трех таксонов наиболее высоким уровнем генетического разнообразия характеризуется A. uralensis. Результаты молекулярного датирования позволяют предположить, что из трех видов первым заселил территорию Центрального Предкавказья A. uralensis в два этапа: примерно 370–350 тыс. лет назад и около 256 тыс. лет назад. Позднее на данную территорию в два этапа проник A. witherbyi: примерно 166–154 тыс. лет назад и 125–115 тыс. лет назад. A. flavicollis является самым молодым вселенцем на Центральном Предкавказье. Его эволюционный возраст составляет примерно 100 тыс. лет. В работе приведены особенности распространения и ландшафтно-биотопического распределения представителей рода Apodemus на исследованных территориях Центрального Предкавказья.

## Introduction

The study of genetic differentiation and phylogeography of widespread species is important for the study of modern biodiversity, the formation of species ranges, the consequences of global climate change and anthropogenic impacts, and issues of biological expansion and invasion (Yalkovskaya et al., 2022). Genetic diversity is considered one of the many important factors determining species adaptation to changing environmental conditions (Soulé, Simberloff, 1986). The higher the genetic diversity of species living in both undisturbed and highly disturbed environments, the more successful the adaptation, the more capable the populations are of evolving in response to the pressure of external environmental factors and the less likely they are to disappear if environmental conditions change (cited in Poliakova, 2022).

Currently, due to anthropogenic pressure and rapid land-use changes, steppe ecosystems are among the most heavily modified habitats. As S.I. Sigida (2010) rightly notes, as a result of human impact, the biogeocenoses of the steppe plains and a significant portion of the forest-steppe of the Central Ciscaucasia have almost completely lost their original appearance over the course of several decades of the 20th century. The planting of shelterbelts, artificial forests, irrigation, and water supply facilitated the expansion of forest and aquatic animal groups into the steppe regions, as well as the emergence of migrants from neighboring regions (cited in Pushkin, 2015). Clearly, such changes impact the species diversity of steppe ecosystems.

It should also be noted that anthropogenic impact results in the reduction of suitable habitats, their fragmentation, etc. Also, as is known, long-term isolation of a population caused by natural or anthropogenic fragmentation of the habitat can lead to a change in the genetic structure of the population, in particular, to a decrease in genetic diversity and a reduction in the total effective population size (Radespiel, Bruford, 2014; Wang et al., 2017).The relevance of this study stems from the lack of modern data on the species composition of members of the genus Apodemus in the steppe zone of the Central Ciscaucasia, obtained through molecular genetic analysis. Existing studies were conducted before the advent of these methods in the second half of the 20th century (Tembotov, 1972). It should be noted that cryptic species, which include members of the genus Apodemus, are virtually indistinguishable based on morphological characteristics. Incorrect identification of biologically important species creates false perceptions of biological diversity (Sukhikh, 2013).

It is also important to note that for some representatives of the genus Apodemus, genetic data are represented by single sequences of the cytochrome b (cyt b) gene of mtDNA. For example, only 18 sequences of the Caucasian wood mouse have been deposited in the international genetic database GenBank, some of which are from Iran. Regarding Apodemus witherbyi Thomas, 1902, the 26 cyt b gene sequences submitted come only from Ukraine and Iran. As can be seen, sequences from the study area are missing from the GenBank database.

In light of the above, it was of interest to study the species composition, genetic structure, and genetic diversity of members of the genus Apodemus in the steppe zone of the Central Ciscaucasia. The selection of members of the genus Apodemus was deliberate: firstly, they constitute a significant proportion of the catches; secondly, they represent species with diverse ecological preferences, which can be found in both natural (untouched virgin steppe areas, intrazonal forests) and anthropogenically transformed landscapes (haystacks in row crop fields, canal banks, shelterbelts, etc.).

## Materials and methods

Tissue samples from 153 Apodemus wood mice collected from various locations in the steppe zone of the Central Ciscaucasia were used in the work.

The study was conducted in the Maysky, Prokhladnensky, and Tersky districts of the Kabardino-Balkarian Republic (KBR) (Fig. 1). In the Maysky district, the study covered biotopes in the vicinity of three settlements (Novoivanovka village, khutor Baksansky, and khutor Novokursky). Due to their proximity, we considered it appropriate to combine them, and this area is designated as khutor Baksansky (N 43.6640° E 043.9362°, h – 224 m above sea level) in this study.

**Fig. 1.**
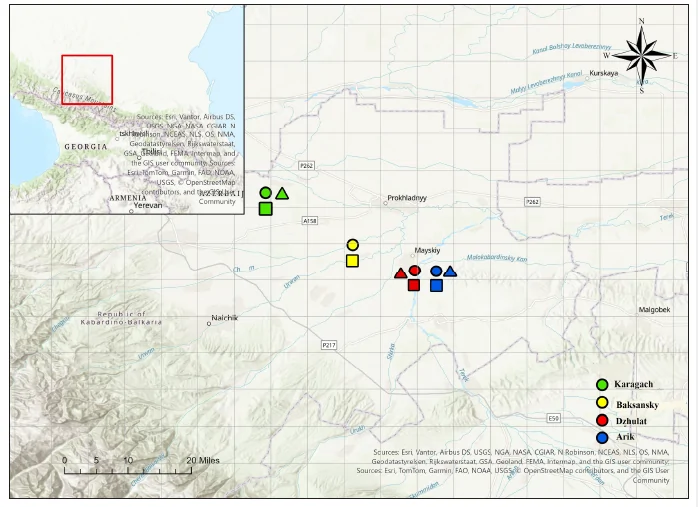
Map of the origin of the material of representatives of the genus Apodemus from the steppe zone of the Central Ciscaucasia: circles – Apodemus uralensis Pallas, 1811, squares – Apodemus flavicollis Melchior, 1834, triangles – Apodemus witherbyi Thomas, 1902.

The material was collected in three biotopes: 1. Forest shelterbelts; 2. Haystacks located in winter crop fields; 3. A boundary directly adjacent to irrigation canals, located between agricultural crop fields.

In the Prokhladnensky district, in the vicinity of the Karagach village (N 43.7838° E 43.7369°, h – 290 m above sea level), the research covered two biotopes: 1. Lowland forest; 2. Meadow located between the road and the agrocenosis. In the Tersky district, the material was collected at two geographical points: in the vicinity of the Dzhulat village (N 43.6050° E 44.0077°, h – 215 m above sea level) – two biotopes: 1. Virgin steppe areas unsuitable for agricultural cultivation; 2. Lowland forest; and in the vicinity of the Arik village (Arik ridge N 43.6036° E 44.1268°, h – 233 m above sea level) – three biotopes: 1. Steppe area: undisturbed forbfescue community on the slope of the ridge; 2. Shelterbelt; 3. Bank of the Small Kabardian irrigation canal.

Tissue samples were fixed in 96 % ethanol and used for manual DNA extraction using the Diatom™ DNA Prep 100 reagent kit (Isogen Laboratory, Moscow) according to the manufacturer’s instructions. Amplification of the cyt b gene was performed by polymerase chain reaction (PCR), using primers from (Yasuda et al., 2005) with the PCR cycle parameters recommended in the cited work. The resulting PCR products were purified by reprecipitation in 0.15 M sodium acetate in 90 % ethanol, followed by washing with 70 % ethanol. The result was visualized by electrophoresis in 1.5 % agarose gel in the presence of ethidium bromide. Sequencing of nucleotide sequences was performed in both directions at Syntol (Moscow).

Alignment of the obtained sequences was performed using the ClustalW module in the BioEdit software package (Hall, 1999). Weighted (net distance) intergroup genetic distances using the Kimura two-parameter model (K2P) (Kimura, 1980) were calculated in the Mega 6 program (Tamura et al., 2013).

The Bayesian algorithm was implemented in MrBayes v3.2.6 using the Markov chain Monte Carlo (MCMC) algorithm (Ronquist, Huelsenbeck, 2003). The Bayesian information criterion (BIC) was used to find optimal models. Nucleotide substitution models were selected using Mega 6. The analysis generated 5×106 Markov chain trees, with tree selection performed every 1,000 generations. The first 25 % were discarded as “burnout”. Phylogenetic trees were visualized using FigTree.

To estimate the divergence time, an additional analysis was performed in BEAST version 1.10.4 (Suchard et al., 2018). For each species, nucleotide substitution models calculated in the Mega 6 program are presented: HKY+G+I for A. uralensis and A. flavicollis, and HKY+G for A. witherbyi. The analysis was performed using an uncorrelated lognormal relaxed molecular clock model and a constant-size coalescence model. The MCMC chain length was 60 million generations, with a sampling step of one thousand and discarding the first 10 % of generations (burn-in); the effective sample size (ESS) for all parameters was greater than 200. Convergence of parameters was confirmed by ESS values >200, estimated in the Tracer 1.7 program (Rambaut et al., 2018).

The calibration points were taken from the work of Y. Amir Afzali and R. López-Antoñanzas (2024), performed on Iranian rodents, including representatives of the Muridae family. According to the data of the noted authors, the species Mus Linnaeus, 1758 and Apodemus Kaup, 1829 diverged from Rattus Fischer, 1803 approximately 12.28 million years ago (mya). The time estimate of 12.28 mya for the root node of divergence between Rattus and Mus + Apodemus was used as the first calibration point. We also used a second calibration point of 2.09 mya – the time of divergence of Mus macedonicus Petrov & Ruzic, 1983 from M. musculus musculus and M. musculus domesticus. Additional calibration points were used for the species A. uralensis and A. flavicollis. For A. uralensis, the time of divergence of the European and Asian races was approximately 1.5 mya (Chelomina, Atopkin, 2010), and 2.6 mya was the time of separation for A. flavicollis and A. f. ponticus (Darvish et al., 2015).

In this study, results were obtained using a mutation rate of 2.4–3.1 % (Suzuki et al., 2015).

The median haplotype network was constructed using Network 4.6.1 using the Median Joining algorithm (Bandelt et al., 1999).

Molecular diversity indices – the number of haplotypes, haplotype (h) and nucleotide diversity (π) values, as well as the Tajima (1989) and Fu (1996) neutrality tests – were calculated using Arlequin v.3.5 (Excoffier, Lischer, 2010).

For genetic diversity assessment, the analyzed sequence length was 960 bp for all three taxa studied.

Information on the original material is provided in in Table S1 in Supplementary Material1, and the data obtained from GenBank can be found in Table S2.

Supplementary Materials are available in the online version of the paper: https://vavilov.elpub.ru/jour/manager/files/Suppl_Tem_Engl_30_6.pdf


## Results

This paper presents the results of an analysis of the variability of the mtDNA cyt b gene fragment in representatives of the subgenus Apodemus from geographical samples from the steppe zone of the Central Ciscaucasia (Arik village, Dzhulat settlement, Karagach village, khutor Baksansky), which have not been previously studied. Molecular genetic analysis yielded 153 nucleotide sequences of the mtDNA cyt b gene fragment, of which 73 belonged to A. uralensis, 37 to A. flavicollis, and 43 to A. witherbyi = A. fulvipectus. Within the alignment, 225 polymorphic sites were detected.

**Genetic characterization of A. uralensis**

Nucleotide sequences of a 960-bp mtDNA cytochrome b gene fragment were obtained for 73 A. uralensis samples. Based on the analysis of 73 mtDNA cytochrome b gene fragment sequences from four A. uralensis populations, 75 polymorphic sites were identified, 40 of which were parsimony-informative.

To construct the phylogenetic tree, in addition to the A. uralensis sequences obtained in this study, another 98 from the GenBank database (948 bp) were included in the alignment. Figure 2 shows the Bayesian tree obtained for 116 A. uralensis haplotypes. As can be seen from the resulting dendrogram, two statistically supported clusters can be identified within the A. uralensis group: A. uralensis 1 and A. uralensis 2, with a distance of 6.2 % (Fig. 2). The first cluster corresponds to the so-called European race, the second, to the Asian one, which is consistent with publications based on the use of karyotypic and biochemical as well as molecular genetic data (Chelomina et al., 2007; Chelomina, Atopkin, 2010). Remarkably, out of the 27 European A. uralensis sequences from the GenBank database included in the analysis, not a single haplotype was identical to the mitotypes of A. uralensis from the steppe Ciscaucasia. Haplotypes of A. uralensis from the Central Ciscaucasia occupy a somewhat isolated position on the phylogenetic tree, but with a low posterior probability.

**Fig. 2.**
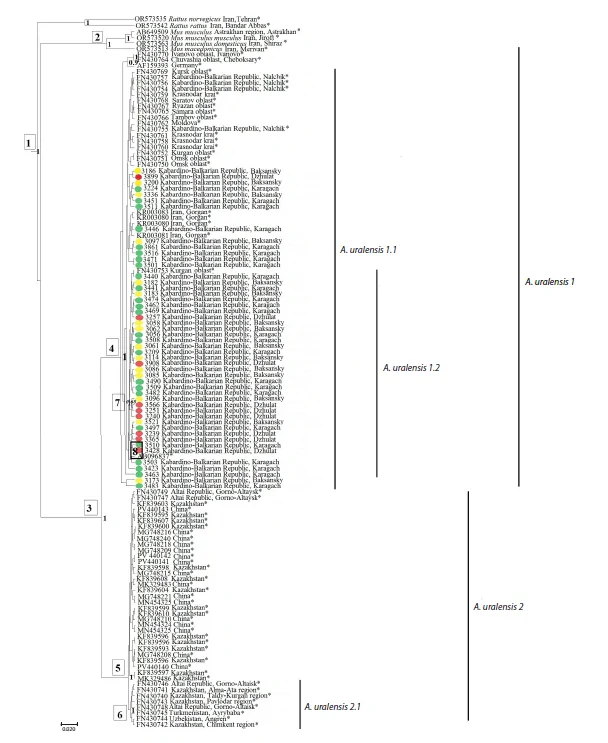
Bayesian phylogenetic tree of A. uralensis based on the analysis of haplotypes of the mtDNA cyt b gene fragment (948 bp). Here and in Figures 4 and 6: numbers at branch nodes are posterior probabilities; numbers in squares are node numbers. Haplotypes from GenBank are marked with an asterisk (*).

Haplotypes of mice from Iran and the Russian Plain also do not form distinct clusters with high posterior probability values, and the presence of mitotypes from Iran and the Kurgan region among haplotypes of mice from the Central Ciscaucasia likely indicates ongoing gene flow. This is also supported by the low genetic distances between mice from the Central Ciscaucasia and Iran (0.12 %), and mice from the Russian Plain (0.26 %).

To achieve more precise clustering, a median network (Fig. 3) was constructed based on 99 European A. uralensis sequences. The median network also demonstrates a lack of clear differentiation between haplotypes of mice from different regions, including those from the GenBank database included in the analysis

**Fig. 3.**
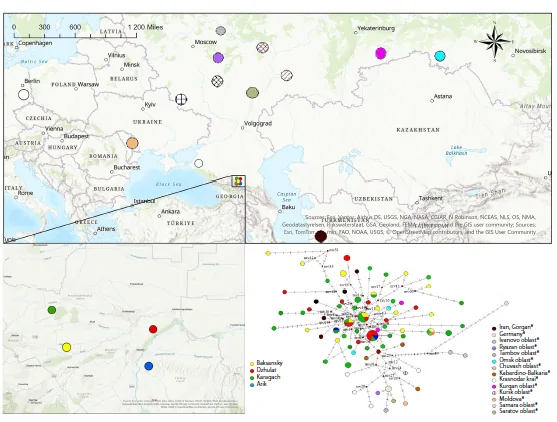
Median haplotype network of A. uralensis based on 73 sequences of the 948-bp cyt b gene fragment obtained in this study and 26 sequences from the GenBank database. Here and in Figures 5 and 7: the circle size is proportional to the number of identical haplotypes; dashes indicate mutations, mv denotes hypothetical haplotypes, and asterisk (*) denotes haplotypes from GenBank.

In the Central Ciscaucasian species A. uralensis, 50 haplotypes were described among 73 sequenced sequences, of which 40 are unique (80 %). The remaining 20 % of haplotypes were found in 2–7 individuals. A characteristic feature of the median network of A. uralensis is the large number of alternative connections that arise as a result of reverse or parallel mutations (Fig. 3).

Genetic distances between geographic samples of A. uralensis from the Ciscaucasian steppe also revealed no differences. The maximum distance (K2D) in a pairwise comparison of four samples (Karagach, Dzhulat, Baksansky, and Arik) did not exceed 0.06 %.

Haplotype diversity was quite high in all samples, with nucleotide diversity ranging from 0.0044 to 0.0089 (Table 1). The lowest values were observed in the sample from Dzhulat, and the highest, in the one from Baksansky. Almost all A. uralensis samples studied showed significantly negative values for the Fu and Tajima tests for evolutionary neutrality.

**Table 1.**
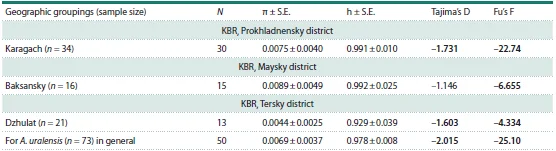
Haplotype (h) and nucleotide (π) diversity indices and Tajima and Fu test values for A. uralensis from the Central Ciscaucasia Notе. Here and in Tables 3 and 5: N – number of haplotypes; S.E. – standard error. KBR – Kabardino-Balkarian Republic. Statistically significant test values are highlighted in bold.

**Divergence time of the main A. uralensis clusters**

According to our calculations, the separation of the two races, A. uralensis 1 and A. uralensis 2, i. e. the European and Asian, occurred approximately 1.475 million years ago (95 % HPD: 0.930–2.076) (Table 2, node 3). Our date does not contradict the data of G.N. Chelomina and D.M. Atopkin (2010), who showed the divergence of the Asian and European races approximately 1.5 million years ago. The most recent common ancestor of the European race of A. uralensis (A. uralensis 1) existed approximately more than 600 thousand years ago (95 % HPD: 0.346–0.951) (Table 2, node 4), and that of the Asian race (A. uralensis 2), approximately 252 thousand years ago (95 % HPD: 0.121–0.424) (Table 2, node 5). The age of the cluster (A. uralensis 1.1), which included almost all haplotypes from the Russian Plain and the Central Ciscaucasia (from FN430769 to 3463), averaged approximately 400 thousand years (95 % HPD: 0.212–0.566) (Table 2, node 7). The age of the haplogroup A. uralensis 1.2, represented only by haplotypes A. uralensis of the Central Ciscaucasia, was approximately 260 thousand years (Table 2, node 8).

**Table 2.**
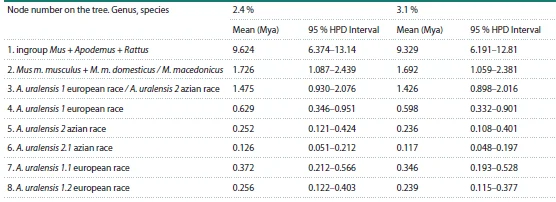
Time of existence (in million years) of the last common ancestor (TMRCA) in the main clades of A. uralensis based on the results of the analysis of differences in cyt b sequences

**Genetic characteristics of A. flavicollis**

The phylogenetic tree obtained from the Bayesian analysis clearly demonstrates the division of A. flavicollis haplotypes into two clusters with high posterior probabilities (Fig. 4). The first cluster, A. flavicollis 1, is formed by haplotypes of mice from Iran, Turkey, Israel, and the Central Ciscaucasia. The second, largest cluster, A. flavicollis 2, includes several haplogroups: A. flavicollis 2.1, 2.2, and 2.3. Haplogroup A. flavicollis 2.1 includes animals from France, Bosnia and Herzegovina, and Belarus, Italy, Greece, Spain, Sweden, Turkey, the Czech Republic, Iberian Peninsula, Germany, Macedonia, Austria, Switzerland, Hungary, Russia (Samara). Haplogroup A. flavicollis 2.2 is represented primarily by haplotypes of mice from Russia (Urals), Ukraine (Chernobyl), Macedonia, Romania, Lithuania, Belarus, Estonia, and France. Haplogroup A. flavicollis 2.3 includes animals from Russia (Voronezh, Urals, Volgograd), Yugoslavia, and Romania. The distance between clusters A. flavicollis 1 and A. flavicollis 2 is 5.3 %. It should be noted that the taxonomic status of A. flavicollis 1, the distribution of which covers Georgia, Dagestan, Krasnodar Krai, Azerbaijan, Armenia, and territories bordering the Caucasus (Turkey and Iran), remains disputed.

**Fig. 4.**
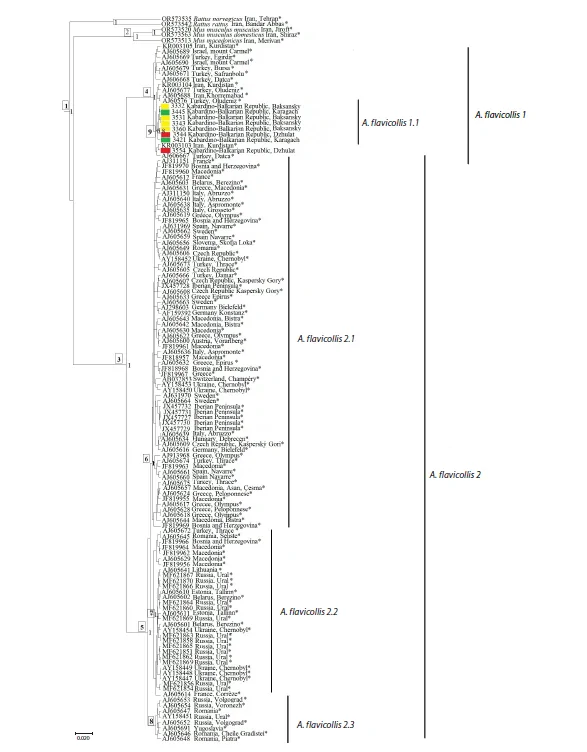
Bayesian phylogenetic tree of A. flavicollis based on the analysis of haplotypes of the mtDNA cyt b gene fragment (870 bp).

The median network (Fig. 5), constructed using additional data on 13 nucleotide sequences of the homologous region of mtDNA of A. flavicollis from Iran, Turkey, and Israel, shows that the haplotypes of mice from the Central Ciscaucasia are represented by one dominant haplotype and a fan of haplotypes with minor frequencies of occurrence, differing from each other by 1–4 nucleotide substitutions, which indicates the absence of subdivision of the samples.

**Fig. 5.**
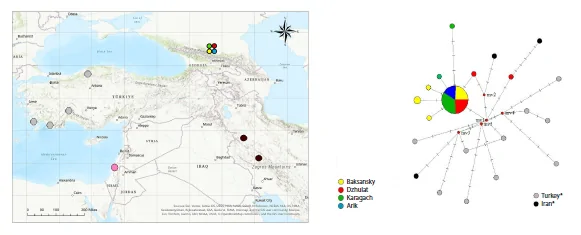
Median haplotype network of A. flavicollis based on 37 cyt b gene fragment sequences obtained in this study and 13 sequences from the GenBank database.

The 50 analyzed mtDNA cyt b gene fragment sequences formed 21 haplotypes. The fragment under study was found to contain 41 variable sites. The dominant haplotype was observed in 29 individuals and was widely represented in all studied localities: Arik, Dzhulat, Karagach, and Baksansky. Of the 37 haplotypes in the identified area, only six were unique, accounting for only 16 %. Overall, the network had a fairly extensive structure with a significant number of median vectors representing hypothetical, likely ancestral haplotypes. No completely identical haplotypes were identified when compared with sequences from Iran and Turkey (Genbank). The genetic distances in pairwise comparison of A. flavicollis samples were zero, indicating that they belong to the same population; however, when compared with the grouping from Iran and Turkey, they were 0.16 and 0.14 %, respectively.

The phylogenetic structure we discovered, characterized by a small number of haplotypes and their close proximity, likely indicates that the study area was populated by a small number of founder individuals originating from a single ancestral population.

Table 3 shows that virtually all samples were characterized by low nucleotide and haplotype diversity.

**Table 3.**
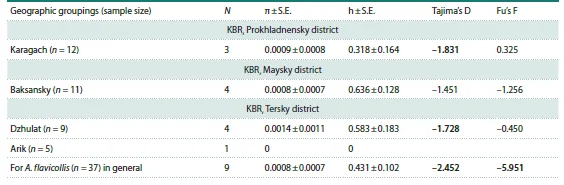
Haplotype (h) and nucleotide (π) diversity indices and Tajima and Fu test values for A. flavicollis from the Central Ciscaucasia

The observed values in the overall A. flavicollis sample were also low. The sample from the village of Arik was characterized by zero values, as it was monomorphic, likely due to its small size. Nearly all samples showed significantly negative values for the Tajima and Fu tests. In the overall sample, both neutrality tests were also significantly negative.

**Divergence time of the main clusters of A. flavicollis**

As can be seen from Table 4, the split between A. flavicollis 1 and A. flavicollis 2 occurred approximately two million years ago (Table 4). The age of the most recent common ancestor of the A. flavicollis 1 cluster, which includes haplotypes from Iran, Turkey, Israel, and the Central Ciscaucasia, is approximately 300 thousand years (95 % HPD: 0.123–0.525) (Table 4, node 4).

**Table 4.**
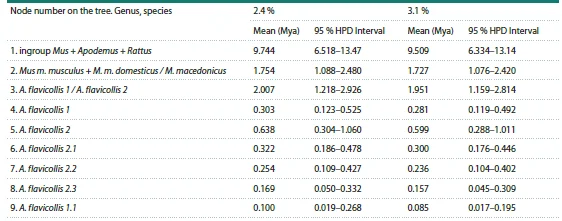
Time of existence (in million years) of the last common ancestor (TMRCA) of the main clades of A. flavicollis based on the analysis of differences in cyt b

The age of the most recent common ancestor of the A. flavicollis 1.1 cluster, represented only by haplotypes of mice from the Central Ciscaucasia, averaged approximately 100 thousand years (95 % HPD: 0.019–0.268) (Table 4, node 9). Within the larger A. flavicollis 2 cluster, which includes haplotypes from all regions of the Western Palearctic, including western (France), northern (Sweden), and eastern Europe to Ukraine, as well as three Mediterranean peninsulas (Spain, Italy, and the Balkans), several groups with different evolutionary ages were distinguished. The age of the A. flavicollis 2 cluster was approximately 640–600 thousand years (Table 4, node 5), and that of haplogroups A. flavicollis 2.1, A. flavicollis 2.2, and A. flavicollis 2.3 ranged from 330 to 160 thousand years (Table 4, nodes 6–8).

**Genetic characteristics of A. witherbyi**

As Figure 6 shows, all of the analysed A. witherbyi haplotypes formed a single large cluster, A. witherbyi 1, which included haplotypes from Iran, Israel, Turkey, and the Central Ciscaucasia. Within this cluster, two haplogroups emerged, represented only by animals from the Central Ciscaucasus: A. witherbyi 1.1 with a low posterior probability value of >0.6 and A. witherbyi 1.2 with a high posterior probability value of 0.9.

**Fig. 6.**
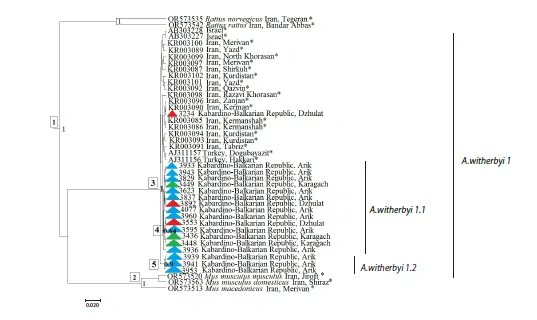
Bayesian phylogenetic tree of A. witherbyi based on the analysis of haplotypes of the mtDNA cyt b gene fragment (870 bp).

The median network of A. witherbyi is characterized by a rather complex structure with a large number of hypothetical haplotypes through which the transition from one haplotype to another could occur. A total of 36 haplotypes have been described, of which two are dominant (Fig. 7). The majority of haplotypes (32 of 36) are unique. The most common haplotype was observed in 21 individuals (48.8 %) from Dzhulat and Karagach, and the second most frequent haplotype was observed in five individuals from the same localities. As can be seen, the distribution of haplotypes in this species, as in others, is not associated with geographic localization.

**Fig. 7.**
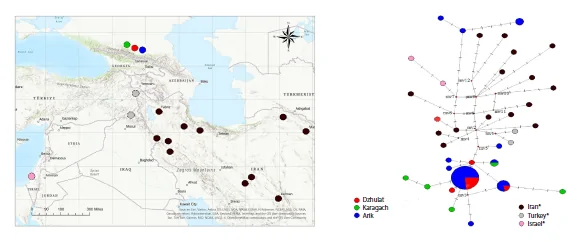
Median haplotype network of A. witherbyi based on 43 cyt b gene fragment sequences obtained in this study and 19 sequences from the GenBank database

As with the previous species, the genetic distances between the studied samples were very low and did not exceed 0.0009. The total sample from the Ciscaucasia differed from the sample from Iran with a distance of 0.0017, from Turkey – 0.0028, from Israel – 0.0035.

Among individual groups, the highest values of both haplotype and nucleotide diversity were observed in A. witherbyi from the vicinity of the village of Karagach (Table 5). The remaining two samples, from the villages of Dzhulat and Arik, were characterized by similar values of haplotype diversity, with higher nucleotide diversity in the latter sample. Overall, low values of both indices were observed for the species: h = 0.759, π = 0.0037.

**Table 5.**
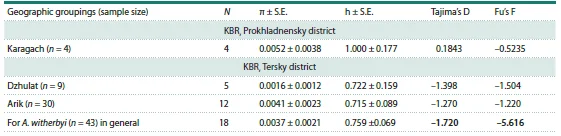
Haplotype (h) and nucleotide (π) diversity indices and Tajima and Fu test values for A. witherbyi from the Central Ciscaucasia

**Divergence time of the main clades of A. witherbyi**

The age of the A. witherbyi 1 cluster, represented by haplotypes from the territory of Israel, Iran, Turkey, and the Central Ciscaucasia, was on average about 350–330 thousand years (95 % HPD: 0.200–0.530 and HPD: 0.184–0.503, respectively) (Table 6, node 3). In the territory of the Central Ciscaucasia, two haplogroups were distinguished: with a low posterior probability value – A. witherbyi 1.1 and with a high one – A. witherbyi 1.2. The most recent common ancestor of haplogroup A. witherbyi 1.1 existed between 166 and 154 thousand years ago (95 % HPD: 0.071–0.292) (Table 6, node 4), and that of haplogroup A. witherbyi 1.2, approximately 125– 115 thousand years ago (95 % HPD: 0.031–0.245) (Table 6, node 5).

**Table 6.**
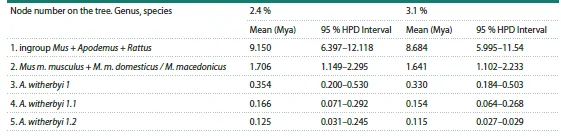
Time of existence (million years) of the last common ancestor (TMRCA) of the main clades of A. witherbyi based on the analysis of differences in cyt b

Biotopic distribution and abundance of representatives of the genus Apodemus in the Central Ciscaucasia

In the Central Ciscaucasia, in the studied steppe regions (Maysky, Tersky, and Prokhladnensky districts), representatives of the genus Apodemus (A. uralensis, A. witherbyi and A. flavicollis) are observed to coexist almost everywhere, sympatrically and in places symbiotically, with the exception of the Maysky district, where A. witherbyi has not been recorded. All three Apodemus species were observed in all the studied biotopes: forest, shelterbelt, banks of an irrigation canal, open habitats, meadow areas (virgin lands), and wastelands between agrocenoses. However, in relation to the study area, along with the symbiotopic habitation of the species, the division of their habitats and the greatest or least gravitation of species to certain biotopes are noted.

The abundance of species varies depending on the type of habitat, year and season. So, the values for A. uralensis range from common to numerous (1–18.1 ind./100 trap-days). It is found in forest shelterbelts with pedunculate oak and on the banks of irrigation canals with a cereal-legume phytocenosis, in the vicinity of khutor Baksansky, Maysky district, where the abundance in some years reaches 8.3 and 10.6 ind./100 trapdays, respectively. In general, the relative abundance of A. uralensis in the studied biotopes was on average (ind./100 trapdays): in the forest – 4.9–9.6; in the shelterbelt – 8.3; on the bank of an irrigation canal – 1.4–6.6; in the meadow – 4.2.

A. flavicollis was recorded in all biotopes of the studied area; in the forests and shelterbelts near khutor Baksansky and Karagach village, the relative abundance of the species reached its maximum values (14 and 14.8 ind./100 trap-days, respectively). The species was common on the banks of irrigation canals with a cereal-legume phytocenosis near khutor Baksansky and Arik village. The average relative abundance values of A. flavicollis were (ind./100 trap-days): in the forest – 2.6–7.6; in the shelterbelt – 8.8; on the bank of an irrigation canal – 1.9–4.9; in the meadow – 1.7.

The third species of the genus Apodemus, A. witherbyi, is present in all studied biotopes of the surveyed areas, with the exception of Maysky district. A. witherbyi is most often found in meadows and virgin lands unsuitable for plowing, with wormwood-grass-forb phytocenoses, where the relative abundance ranges from 1.7 to 4.2 ind./100 trap-days. However, in the forest shelterbelt around Arik, its abundance reaches its highest levels

## Discussion

Genetic structure of representatives of the genus Apodemus

Based on an analysis of the mitochondrial DNA cyt b gene fragment variability, the species composition and genetic diversity of sibling species of wood mice of the genus Apodemus in the steppe zone of the Central Ciscaucasia were studied for the first time. Molecular genetic methods revealed the presence of three species of the genus in the designated area: A. uralensis, A. flavicollis and A. witherbyi, with genetic distances ranging from 10 to 13 %. The maximum level of differences was observed when comparing A. witherbyi with two other taxa: A. uralensis and A. flavicollis. Intraspecific genetic distances among the studied taxa were minimal, not exceeding 0.06 %.

Among the three species, A. uralensis exhibits the greatest genetic diversity (Table 1). A. witherbyi occupies an intermediate position (Table 5), while A. flavicollis exhibits the lowest level of molecular genetic variability among the studied species (Table 3). Inclusion of sequences of suitable length from the GenBank database in the analysis revealed no completely identical haplotypes in all three taxa studied.

For comparison, we note that in previously studied A. flavicollis samples from the Western and Eastern Caucasus, haplotype diversity ranged from 0.867 to 0.978, and nucleotide diversity, from 0.0044 to 0.0051, while in the combined sample, these values were 0.918 and 0.0050, respectively (Amshokova et al., 2024). The haplotype and nucleotide diversity values in the combined sample of A. flavicollis from the Central Ciscaucasia are lower than those obtained previously. Negative and significant values of the Tajima and Fu tests observed in the combined sample provide evidence of population expansion of A. flavicollis. The dominant haplotype of A. flavicollis is found in 78.3 % of individuals across all geographic samples. This median network structure, as well as genetic diversity indicators, likely indicate a recent colonization of the studied area by a limited number of founder individuals carrying the dominant haplotype. The absence of genetic differences between the four samples also serves as evidence of a common origin.

A. uralensis, unlike A. flavicollis, is characterized by a more complex haplotype network structure and the highest values of the haplotype and nucleotide diversity indices (Fig. 3). In this case, we observe the presence of a large number of alternative links of varying lengths, which are the result of back or parallel mutations, leading to the formation of multiple closed loops. In A. uralensis, as in other species, the haplotype distribution does not demonstrate a clear, reliable relationship with the geographic location of the samples. This network structure, along with high values of haplotype and nucleotide diversity, indicates long-term population evolution under stable conditions, likely indicating significant gene flow with neighboring areas.

A comparison of the level of genetic variability of A. uralensis with known literature data suggests a relatively high level of genetic diversity characteristic of the species as a whole. Similar results were obtained by other authors when analyzing the cyt b gene region of A. uralensis in the Kabardino-Balkarian Republic – h = 1.000, π = 0.0131; Krasnodar Krai – h = 0.900, π = 0.0079 (Chelomina, Atopkin, 2010); and the Lower Don – h = 0.933, π = 0.0056 (Stakheev, 2024).

The combined sample of A. witherbyi occupies an intermediate position in terms of genetic variability among the studied representatives of the genus Apodemus. Compared with data reported in the literature, the species in the study area is characterized by lower values of genetic diversity. So, according to data from N. Shad et al. (2016), in the combined sample of A. witherbyi from Iran, the haplotype diversity was 0.914, and the nucleotide diversity was 0.0043. The relatively low genetic diversity of A. witherbyi from the Central Ciscaucasia is likely due to its late introduction to the study area.

Divergence time of the main clades of representatives of the genus Apodemus

Speciation within the subgenus Sylvaemus occurred between 1.2 and 5.7 million years ago, i. e. in the Pliocene-Pleistocene, and its divergence from the genus Apodemus occurred more than 7.5 million years ago, i. e. in the Miocene (Chelomina, Suzuki, 2006).

According to the results of our molecular dating, the separation of the two races of A. uralensis – European and Asian – occurred approximately 1.5 million years ago (95 % HPD: 0.929–2.076) (Fig. 2, Table 2), which is consistent with the data of G.N. Chelomina and D.M. Atopkin (2010).

The divergence time between A. flavicollis 1 (Fig. 4), which is distinguished by some authors as an independent species (A. ponticus (Bogdanov et al., 2012)), and by others as a subspecies (A. flavicollis ponticus (Gromov, Erbaeva, 1995)), and A. flavicollis 2 (Fig. 4) is about two million years. Our data are close to the results of foreign authors (Michaux et al., 2004), who showed the separation of clade 1 (Turkey, Iran, Israel) and clade 2, which includes all populations of the Western Palearctic, from the Iberian Peninsula in the west to the south of Russia (Samara) in the east and Estonia in the north, 2.2–2.4 million years ago. According to J.R. Michaux et al. (2004), this tree structure can be explained by the isolation of two A. flavicollis groups in two different refugia: one in the Middle East and the other in the Balkan region during one of the first Quaternary climatic fluctuations. The authors believe that the entire Western Palearctic was recolonized not from the Middle Eastern, but from the Balkan population, a finding they tentatively attributed to the presence of strong biogeographic barriers, such as the Black Sea and the Caucasus, which prevented contact between Palearctic and Middle Eastern animals

According to literature data, the divergence between A. witherbyi and other closely related species occurred 6 million years ago (Darvish et al., 2015).

Before discussing the data obtained on the evolutionary age of Apodemus mice in the Central Ciscaucasia, it is necessary to highlight some key points in the evolution of the Caucasus itself, which forms the isthmus between Europe and Asia. According to the literature, approximately 1.8–0.7 million years ago, during the Eopleistocene (Middle Pleistocene), the Caucasus was a peninsula. During this period, it was washed by the Absheron Sea, which existed in the territories of today’s Black Sea, Azov, Caspian, Aral, lower Volga regions, Turkmenistan, and Kazakhstan. During this period, fauna, including mammals, was closely connected with that of Asia Minor via Transcaucasia. The formation of the isthmus resulted from the repeated merging of the Pontus and Caspian basins during the Pleistocene. So, A.A. Svitoch and V.M. Sobolev (2011), while studying the Pleistocene Manych Straits, which is the natural boundary between the Russian Plain and the North Caucasus, identified the following periods of merging of the Pontus and Caspian Seas during the Pleistocene.

1. In the second half of the Early (Lower) Pleistocene (Gelazian: 2.58–1.80 million years ago, according to the Russian stratigraphic scale approved in March 2024), the Baku (Late Chaudinian) Strait existed, through which the waters of the Baku transgression of the Caspian Sea discharged into the Late Chaudinian basin of Pontus. 2. In the middle of the Middle Pleistocene (Eopleistocene, 1.80–0.781 million years ago), a second junction of the Caspian and Black Sea basins was noted, resulting in the formation of the Early Khazar Strait, which was caused by the high level of the Early Khazarian transgression of the Caspian Sea and the overflow of its waters into Pontus. 3. At the beginning of the Late (Upper) Pleistocene (Neopleistocene, 0.781–0.129 million years ago), the Late Khazar (Karangat) Strait emerged in the Manych Depression, caused by the major Karangat transgression of the Pontus and the relatively small Late Khazar transgression of the Caspian. 4. In the middle of the Late Pleistocene, after the regression of the Karangat and Late Khazar basins, a vast freshwater reservoir existed in the Manych Depression. It occupied the entire lowland portion of the depression, filling the troughs of the ancient straits. Compared to the preceding Late Khazar (Karangat) Strait, the lake had a larger area and depth

Summarizing the presented data on the paleogeography of Pontus, the Caspian Sea and Manych, it can be assumed that the penetration of mammal fauna from the Russian Plain to the North Caucasus periodically occurred in the Neopleistocene starting from 780 thousand years ago.

No less interesting and important, in addition to the history of the formation of the modern territory of the Caucasus, are the paleontological data of A.K. Markova (2016). The author studied the fauna of small mammals in Europe, including Eastern Europe, in the first half of the Middle Pleistocene in the range of 0.76–0.42 million years ago, and then in the second half in the range of 0.424–0.13 million years ago (Markova, 2017). It should be clarified here that, according to the Russian stratigraphic scale, these intervals correspond to the Late Pleistocene (Neopleistocene). According to the author, in the first half of the Middle Pleistocene, remains of mice of the genus Apodemus from 26 sections in European Russia and Belarus were found in only two sections: Apodemus cf. flavicollis in Belarus (Korchevo locality), and remains of Apodemus ex gr. sylvaticus in Tambov Oblast (Kuznetsovka locality). Of the 35 sections studied in the European part of Russia during the second half of the Middle Pleistocene (~0.424–0.13 million years ago, according to the author), remains of Apodemus ex gr. sylvaticus were found in only one section (the Chekalin locality, Tula Oblast). It is worth clarifying here that, according to modern taxonomy, A. uralensis inhabits the European part of Russia (Gromov, Erbaeva, 1995) and the North Caucasus (Tembotova, 2015). Based on the data of A.K. Markova (2016, 2017), it can be concluded that mice of the genus Apodemus were not widespread or numerous in the European part of Russia during the Middle Pleistocene (in sections, both species were represented by an insignificant number of remains). This is also consistent with the data of N.K. Vereshchagin (1959). The author claimed that paleontological data from Pleistocene deposits in the Caucasus are absent. For example, Apodemus agrarius Pallas (1771) is known in the Caucasus only from Upper Quaternary deposits of the lower Don. The author does not provide any information on other species of the genus, nor does she provide data on representatives of two other closely related genera: Rattus and Mus of the family Muridae Illiger (1811).

Thus, the paleontological data of A.K. Markova (2016, 2017) on small mammals of the European part of Russia allow us to assume that only in the middle of the Late Pleistocene (Neopleistocene), less than 400 thousand years ago, corresponding to the optimum of the Likhvin interglacial (350–300 thousand years ago), did the possibility arise for the penetration of European fauna into the Ciscaucasia from the Russian Plain.

Turning to the evolutionary age of the three mouse species (A. uralensis, A. witherbyi and A. flavicollis) from the study area, the following can be noted in the context of the above. As reflected in the results, the A. uralensis haplotypes from the Central Ciscaucasia do not form a distinct cluster with a high level of support; however, some structure is evident. Therefore, the age was calculated for a cluster combining haplotypes from the Central Ciscaucasia and the Russian Plain, which also included three haplotypes from Iran. The formation time of this mitochondrial clade of A. uralensis is approximately 370–350 thousand years ago (Fig. 2, node 7), corresponding to the so-called Middle Neopleistocene. The cluster represented solely by A. uralensis haplotypes from the Central Ciscaucasia (Fig. 2, node 8) is younger, dating to approximately 256 thousand years ago, corresponding to the Late Neopleistocene. Considering the fact that an insurmountable water barrier regularly appeared between the Russian Plain and the North Caucasus in the Manych region, the little wood mouse penetrated the North Caucasus in at least two stages.

The age of the most recent common ancestor (MRCA) of haplogroup A. witherbyi 1.1 in the Central Ciscaucasia falls between 166 and 154 thousand years, while that of haplogroup A. witherbyi 1.2 falls between 125 and 115 thousand years. The absence of cyt b gene sequences in the GenBank database from other areas of A. witherbyi distribution precludes any speculation about the possible routes of this species’ introduction into the Central Ciscaucasia. Based on the data obtained, the species most likely also entered the North Caucasus in two stages: the late Neopleistocene and early Holocene.

According to data obtained for the Caucasian wood mouse, the age of the most recent common ancestor of haplogroup A. flavicollis 1, represented only by haplotypes of mice from the Central Ciscaucasia, is approximately 100–85 thousand years. These data indicate that A. flavicollis is the youngest invader to the Central Ciscaucasia, with an evolutionary age corresponding to the Middle Holocene. mDNA variability indicates a close relationship with representatives from Asia Minor, suggesting that the mouse entered the Caucasus via the Black Sea coast from Turkey, which is consistent with paleontological data from A.S. Tesakov (2021) on the Black Sea coast.

**Distribution and habitat distribution of representatives of the genus Apodemus**

According to I.V. Zagorodniuk et al. (1997), the range of A. witherbyi extends north from the Main Caucasus Range, to the Don and the Kuma-Manych Depression, and is known throughout the area from the Black Sea (Don, Kuban) to the Caspian Sea (Kuma, Terek). Regarding the yellow-necked mouse, which inhabits the Caucasus, there are two points of view: some authors distinguish it as an independent species, others as a subspecies. Sylvaemus flavicollis ponticus is endemic to the Caucasus Mountains and is common in the broadleaved forests of the Ciscaucasia, Caucasus, and Transcaucasia (Pavlinov, Lisovsky, 2012). As for A. uralensis, electrophoretically typed specimens have been described almost everywhere in the Caucasus and Transcaucasia (Vorontsov, 1992), as well as in the European part of Russia (Chelomina, Atopkin, 2010; Stakheev et al., 2011).

There are differing opinions regarding the biotopic preferences of the studied species. According to E.I. Lashkova and I.I. Dzeverin (2004), A. uralensis is the most polymorphic species, the biotopic and geographic distribution of which is difficult to characterize. Biotopic preferences vary slightly depending on the region. It is believed that in some parts of its range, A. uralensis clearly avoids forests, preferring open habitats such as dry shrublands and even virgin steppe areas. In others, on the contrary, it prefers forests and moist areas with dense vegetation, avoiding dry, open spaces. One of the factors determining the species’ habitat distribution in the Caucasus is humidity. The species avoids dry habitats, giving way to S. arianus (steppe mouse) = A. witherbyi. It is found in lowland and mountain forests, meadows, reed beds along water bodies, and in gardens and vegetable plots (https://biodiversity. ru). It is abundant in the dark coniferous mountain forests of the Caucasus on rocky scree, where it lives in symbiosis with Chionomys gud. Previous studies conducted in the North Caucasus also showed that of the three wood mouse species inhabiting the study area – A. uralensis, A. witherbyi, and A. flavicollis – the former is the most widespread, occurring in all studied biotopes, with the exception of the xerophytic landscapes of the protected areas of the Eastern Caucasus and Rostov Oblast, where the species has not been recorded (Gudova et al., 2021; Amshokova et al., 2024). The range of A. uralensis extends in altitudinal, longitudinal, and latitudinal directions.

A. uralensis is widespread in the steppe regions of the Central Ciscaucasia, inhabiting all studied biotopes. The species accounts for a significant proportion of catches: of 153 genetically dated specimens, 72 (47 %) belonged to this taxon. It prefers forested areas; more than half (approximately 65 %) were captured in the oak-poplar forest near the village of Karagach, where the highest numbers are recorded. The species is also abundant in the maple-elm forest near the village of Dzhulat. At the same time, A. uralensis avoids xerophytic areas; in all the years of research in the Central Ciscaucasia, it was recorded once in a steppe grass-forb biotope (meadow) adjacent to an agrocenosis, in the vicinity of the village of Karagach.

Sympatric and almost universal symbiotopic habitation is noted with A. uralensis and A. flavicollis. Although A. flavicollis is present in all regions of the Central Ciscaucasia, its most preferred biotopes are lowland forests and shelterbelts, consisting of mixed stands of common oak and poplar, where a significant portion of the animals (25 %) were collected in the vicinity of khutor Baksansky and Karagach village. Like A. uralensis, the Caucasian wood mouse avoids meadows and virgin steppe areas adjacent to agrocenoses; a single individual was recorded near the village of Dzhulat, in an open station – a virgin area between agrocenoses. In general, A. flavicollis is a mesophilic species, the optimum range of which is confined to beech forests (Amshokova et al., 2024).

According to a number of authors, the most typical biotope for A. flavicollis is a broad-leaved forest, where the seeds of beech, hornbeam, oak, and some shrubs make up the bulk of its diet; it also reaches high numbers in mountain beech forests and inhabits mixed forests in the presence of broad-leaved species (Popov, 1960; Mammalian…, 1963; Tatarinov, 1973; Pavlinov, 2002). In the Caucasus, A. flavicollis is also found in broad-leaved forests; in the Alazani Valley, it has also been recorded in dry small-leaved forests (Vorontsov et al., 1992); in Krasnodar Krai and the Republic of Adygea, the Caucasian wood mouse gravitates toward low-mountain, highly productive, nut-bearing broad-leaved forests (beech, hornbeam, oak, chestnut) (Okulova et al., 2007).

A genetic study of mice in the North Caucasus also revealed that A. flavicollis is confined to forested (broadleaf) landscapes of the Western and Eastern Caucasus. The species has not been found in the foothills and mid-mountains of the Central Caucasus (Amshokova et al., 2024).

Although A. witherbyi is present in all the studied biotopes of the Tersky District (vicinity of Arik village and Dzhulat settlement) – where it reaches high population densities in shelterbelts – in the Prokhladnensky District (vicinity of Karagach village), it is recorded exclusively in open habitats, such as meadows, and has not been observed in forests. The species accounts for 28 % of the total catch. It is frequently associated with dry habitats.

In the Ciscaucasia, A. witherbyi inhabits open and forestedge biotopes, fields, and in the North Caucasus and Transcaucasia, steppe habitats, including steppe areas, along the edges of broad-leaved forests, and shrub thickets (Zagorodniuk et al., 1997; Stakheev, 2024). Based on our previously obtained data, we also concluded that A. witherbyi is a stenobiont inhabiting xerophytic landscapes (steppe, semi-desert) of the Eastern Caucasus and on the southern border of the Russian Plain (Rostov Nature Reserve) (Gudova, 2021; Amshokova et al., 2024). We recorded the species in the Tlyaratinsky Nature Reserve at an altitude of 1,800 m above sea level, where it lived sympatrically and symbiotopically with A. uralensis, inhabiting a variety of biotopes: rocky slopes with a forb-clover community and oak areas with sparse herbaceous vegetation, cereal-forb biotopes, forest edges and birch forest clearings (Amshokova et al., 2024; Gudova et al., 2026).

## Conclusion

The species composition and genetic diversity of representatives of the genus Apodemus in the steppe zone of the Central Ciscaucasia were studied. Three cryptic species were identified in the designated area: A. uralensis, A. witherbyi and A. flavicollis, with distances ranging from 10 to 13 %. For the study area, the species A. flavicollis and A. witherbyi were described for the first time based on genetically dated material. A. flavicollis and A. uralensis were recorded in all four studied locations: the vicinity of the Arik and Karagach villages, the village of Dzhulat, and khutor Baksansky. A. witherbyi was found in three out of the four geographical locations (the vicinity of the Arik and Karagach villages, and the village of Dzhulat).

Considering the general features of distribution, landscapebiotopic distribution and abundance of representatives of the genus Apodemus, it can be concluded that in the conditions of the steppe zone of the Central Ciscaucasia, A. uralensis, having a wide distribution, is most attracted to forest areas, reaching maximum abundance here, less often – to forest shelterbelts; the species is also numerous in the coastal phytocenoses of irrigation canals, avoiding the most xerophytic meadow stations – virgin wastelands adjacent to agrocenosis. A. flavicollis shares a symbiotic relationship with A. uralensis. It is also most often found in forests and shelterbelts. In addition, it is recorded in moist areas, such as along canal banks, but is absent from open steppe areas, slopes, and wastelands. The habitats of A. uralensis and A. flavicollis are most similar. A. witherbyi exhibits a narrower range than the other two species, preferring open habitats, meadow areas, the banks of irrigation canals, and virgin lands and wastelands, where it is common. However, its highest populations are found in shelterbelts.

A. uralensis is most often dominant in forests, whereas A. flavicollis is a codominant species relative to the year and season studied. Sometimes, a shift in dominance is observed, with the second species predominating. A. witherbyi is most often the absolute dominant species, and less frequently codominant with A. uralensis in meadows and virgin, uncultivated areas. In isolated cases, it is monodominant or codominant with A. flavicollis in shelterbelts and along the banks of irrigation canals.

A. uralensis was the first to penetrate into the Central Ciscaucasia from the Russian Plain in geological time, likely in two stages. The first occurred approximately 370–350 thousand years ago, and the second, approximately 256 thousand years ago, which, according to the latest version of the Russian stratigraphic scale (2024), corresponds to the mid-Pleistocene. This period coincides with the optimum of the Likhvin interglacial. The obtained data on the genetic diversity of A. uralensis suggest that gene exchange with individuals from European Russia is actively ongoing.

A. witherbyi likely also entered the Central Ciscaucasia in two stages: 1) the late Neopleistocene, approximately 166–154 thousand years ago; 2) the early Holocene, approximately 125–115 thousand years ago. It would be premature to determine the species’ origin in the Caucasus due to a lack of material from other regions.

A. flavicollis is the youngest invader in the Central Ciscaucasia. Its evolutionary age is approximately 100 thousand years; the species is a representative of the Holocene fauna in the region. Variability in mDNA indicates a close relationship with species from Asia Minor, suggesting that the mouse entered the Caucasus via the Black Sea coast from Asia Minor.

## Conflict of interest

The authors declare no conflict of interest.
